# Sarcopenia and adipose tissue evaluation by artificial intelligence predicts the overall survival after TAVI

**DOI:** 10.1038/s41598-024-59134-z

**Published:** 2024-04-17

**Authors:** Matej Pekař, Otakar Jiravský, Jan Novák, Piotr Branny, Jakub Balušík, Daniel Daniš, Jan Hečko, Marek Kantor, Robert Prosecky, Lubomir Blaha, Radek Neuwirth

**Affiliations:** 1 Hospital AGEL Třinec-Podlesí, Konská 453, 739 61 Třinec, Czech Republic; 2 Department of Physiology, Faculty of Medicine, Masaryk University, Kamenice 5, 625 00 Brno, Czech Republic; 3 2nd Department of Internal Medicine, St. Anne’s University Hospital in Brno, Pekařská 53, 656 91 Brno, Czech Republic; 4 Faculty of Medicine, Masaryk University, Kamenice 5, 625 00 Brno, Czech Republic; 5 Faculty of Medicine, Palacky University, Krizkovskeho 511/8, 779 00 Olomouc, Czech Republic; 6 The Jackson Laboratory for Genomic Medicine, 10 Discovery Drive, Farmington, CT 06032 USA; 7 Faculty of Electrical Engineering and Computer Science, VSB - Technical University of Ostrava, 17. Listopadu Street 2172/15, 708 00 Ostrava, Czech Republic

**Keywords:** Sarcopenia, Artificial intelligence, Visceral adipose tissue, Subcutaneous adipose tissue, Survival, TAVI, Cardiac device therapy, Interventional cardiology, Risk factors

## Abstract

Sarcopenia is a serious systemic disease that reduces overall survival. TAVI is selectively performed in patients with severe aortic stenosis who are not indicated for open cardiac surgery due to severe polymorbidity. Artificial intelligence-assisted body composition assessment from available CT scans appears to be a simple tool to stratify these patients into low and high risk based on future estimates of all-cause mortality. Within our study, the segmentation of preprocedural CT scans at the level of the lumbar third vertebra in patients undergoing TAVI was performed using a neural network (AutoMATiCA). The obtained parameters (area and density of skeletal muscles and intramuscular, visceral, and subcutaneous adipose tissue) were analyzed using Cox univariate and multivariable models for continuous and categorical variables to assess the relation of selected variables with all-cause mortality. 866 patients were included (median(interquartile range)): age 79.7 (74.9–83.3) years; BMI 28.9 (25.9–32.6) kg/m^2^. Survival analysis was performed on all automatically obtained parameters of muscle and fat density and area. Skeletal muscle index (SMI in cm^2^/m^2^), visceral (VAT in HU) and subcutaneous adipose tissue (SAT in HU) density predicted the all-cause mortality in patients after TAVI expressed as hazard ratio (HR) with 95% confidence interval (CI): SMI HR 0.986, 95% CI (0.975–0.996); VAT 1.015 (1.002–1.028) and SAT 1.014 (1.004–1.023), all *p* < 0.05. Automatic body composition assessment can estimate higher all-cause mortality risk in patients after TAVI, which may be useful in preoperative clinical reasoning and stratification of patients.

## Introduction

Transcatheter aortic valve implantation (TAVI) represents the minimally invasive and highly effective endovascular procedure that enables the replacement of the dysfunctional aortic valve without the need for open-chest operation and is thus especially suitable for frail elderly patients^1^. Studies from recent years show that alterations in body composition parameters determined from the pre-procedural CT scans can be of prognostic significance in this patient group. These parameters relate to the amount, distribution, and/or activity of the skeletal muscle mass and adipose tissue and include skeletal muscle and adipose tissue areas indexed to body heights (i.e., skeletal muscle index–SMI, visceral [VAT] and subcutaneous [SAT] adipose tissue indexes) expressed in cm^2^/m^2^ or their X-ray attenuations (“density”) expressed in Hounsfield units (HU).

The presence of sarcopenia (i.e., age-related loss of skeletal muscle mass and function) or obesity (i.e., excessive accumulation of body fat), or their combinations (so-called “sarcopenic obesity”) affect the clinical course and prognosis of the patients after TAVI. Indeed, sarcopenia is very common in the elderly population (11 to 50% of patients over 80 years based on the methodology used)^2,3^, and the presence of sarcopenia was repeatedly associated with prolonged hospitalization and increased mortality in TAVI patients^4–6^. On the other hand, the presence of obesity, another widespread condition in the elderly population, was shown to be protective in this patient group if defined using body mass index (BMI)^7^—this fact is described as the “obesity paradox.” Nevertheless, recent studies implementing data derived from preprocedural CT-scan are not so conclusive as some of them showed no effect of fat area on mortality^8,9^, while others indicated that alterations not in the adipose tissue area but in the adipose tissue density may be of prognostic significance^10^.

Despite the progress of our understanding of the effects of body composition on patients’ outcomes after TAVI, preprocedural CT scans are still not routinely used in preoperative patient risk stratification or decision-making^11^. Implementation of novel strategies, such as automated CT scan segmentation software, that immediately provide clinicians with prognostic data obtained from already performed CT scans is highly anticipated. Moreover, data are lacking describing the gender differences as only a small number of studies performed gender-specific analysis. Thus, in the current study, we used artificial intelligence software^12^ to segment the CT scans from lumbar 3 (CTL3) vertebra levels^13^ to assess skeletal muscle, visceral and subcutaneous adipose tissue areas, and densities as the parameters of body composition and we have studied their effects on outcomes of the patients undergoing TAVI in the Hospital AGEL Třinec-Podlesí. We show that in males, SMI, together with VAT and SAT densities, predict all-cause mortality. In contrast, only VAT density show trend for predicting all-cause mortality in women. Ultimately, we provide dichotomization of the obtained analysis to stratify the patients as low- or high-risk based on the VAT density values.

## Methods

### Study population

This retrospective study used data from the Registry of patients after transcatheter aortic valve implantation (TAVI Registry), a prospective monocentric registry of patients who have undergone TAVI at Hospital AGEL Třinec-Podlesí, Czech Republic. The Registry was established in 2010 and collects demographic characteristics based on self-reported questionnaires and imaging data, including CT scans obtained before TAVI and clinical characteristics obtained from electronic health records in the follow-up visits. Our study included all registry participants with available demographic characteristics and pre-procedural torso CT scans. All personal data are processed in accordance with the GDPR, and all participants provided written informed consent before enrollment into the Registry. Before undergoing the TAVI procedure, all patients provided written informed consent not only for the procedure itself but also for collecting and using anonymized data for future research purposes. The ethical considerations of using this data for retrospective analysis were thoroughly reviewed and approved by the Hospital AGEL Trinec-Podlesi ethics committee (EK 301/22) according to the principles of the Declaration of Helsinki. The study is registered under the trial number NCT05672160 at the https://ichgcp.net.

We extracted the following subject characteristics from the Registry: sex (male and female), body weight (kg) and height (m), date of birth, and date of TAVI procedure. Furthermore, we extracted data regarding the subject’s personal history, including the presence of primary hypertension, diabetes mellitus, myocardial infarction, and respiratory disease. The subject’s age was calculated as the period between the birth and the date of the TAVI procedure in days. BMI was standardized as $BMI = \frac{W}{{h^{2} }}$, where *W* is the body weight in kilograms and *h* is the subject’s height in meters.

The primary outcome of this study was overall survival (OS), defined as the number of days between the TAVI procedure and the date of death from any cause. For the subjects who died, the time of censoring was defined as the date of death. The survival time was censored on December 31, 2022.

### TAVI performance

Based on the current guidelines, patients were indicated for TAVI due to severe aortic stenosis. TAVI was performed mainly from the transfemoral approach under the sciascopy control, with patients being carefully monitored. Detailed information about the indications for TAVI, routine and alternative approaches to TAVI, and its performance and complications can be found in our previous article^14^. Details about the TAVI procedure from our center and procedural complications are shown in Tables S1 and S2.

### Image acquisition and automated body composition analysis

The CT scans performed before TAVI were extracted from the picture archiving and communication system (PACS). Siemens Somatom Definition (before 2016) and Siemens Somatom Drive (after 2016) were used as CT scanners with a tri-iodinated non-ionic monomeric contrast medium (Iomeron) with ECG gating for the chest area. The thickness of the slices was 3 and 5 mm in the area above and below the diaphragm, respectively. The voltage was 100 kV, and the current was 284 and 84 mAs in the area above and below the diaphragm, respectively, and these values were unchanged for the whole time. The arterial phase cross sections at the third lumbar vertebra (L3) level, where both transverse processes are visible, were identified by operators skilled in radiologic anatomy and exported in DICOM format. We segmented the cross sections using AutoMATiCA, an automated body analysis framework based on neural networks, to build a segmentation map based on L3 CT scans without further editing needed (accessed on Jan 2023 from the published source code repository)^12^. AutoMATiCA is a UNET network and has demonstrated excellent accuracy for biomedical image segmentation. The data set of 893 patients in the AutoMATiCA study was divided so that 80% of scans were used for training the network, 10% for validation during training, and 10% for testing final network accuracy. In the testing cohort, DSC scores indicated excellent agreement between human and network-predicted segmentations. Network segmentation took ~ 350 ms/scan using modern computing hardware^12^.

**Figure 1:**
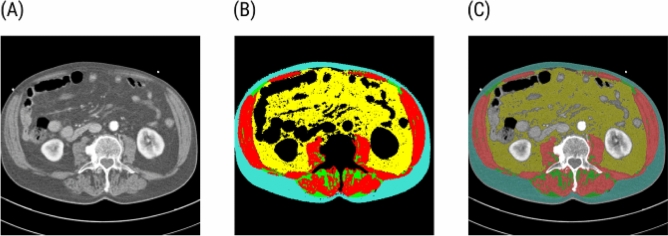
Segmentation of the CT L3 scan cross-section. (**A**) An example of a raw CT cross-section at the third lumbar vertebra level. (**B**) The segmentation map consists of the skeletal muscle tissue (red), intramuscular adipose tissue (green), visceral adipose tissue (yellow), and subcutaneous adipose tissue (turquoise). The black areas are excluded from the analysis. (**C**) An overlay of the segmentation map with the CT cross-section.

The segmentation split the CT scan into several sections: skeletal muscle, adipose tissue, and unsegmented area. At the L3 level, the skeletal muscle tissue included the psoas muscle, quadratus lumborum, erector spinae, external and internal obliques, transversus abdominis, and rectus abdominis. The adipose tissue was further divided into intramuscular adipose tissue, visceral adipose tissue, and subcutaneous adipose tissue (Fig. 1).

For the downstream analysis, the software calculated the areas in cm^2^ and each section’s mean radiation attenuation in Hounsfield units (HU). We used the areas and the attenuation values to calculate the CTL3 parameters. The skeletal muscle index was defined as $SMI = \frac{{S_{sm} }}{{h^{2} }}$ where *S*_*sm*_ was the total area of the skeletal muscles, and *h* was the subject’s height. Similarly, the intramuscular adipose tissue index (IMAT index), visceral adipose tissue index (VAT index), and subcutaneous adipose tissue index (SAT index) were calculated as the respective area normalized by squared body height. The units of all indices were cm^2^/m^2^. Last, we used the mean radiation attenuation values of skeletal muscles as well as of the IMAT, VAT, and SAT as densities. The densities were not transformed; the HU was negative for all adipose tissues.

### Statistical analysis

Statistical analysis was performed using R programming language in the integrated development environment R Studio. Results with a *p*-value below 0.05 were considered statistically significant. Continuous variables were presented as median (interquartile range). Categorical variables were presented as numbers with percentages. We tested the correlation between variables using the Spearman correlation coefficient. Univariate and multivariable Cox proportional hazards models were used to assess the relation of variables with all-cause mortality. Multivariable models were adjusted for age, sex, and presence of comorbidities, including hypertension, diabetes mellitus, history of myocardial infarction, and respiratory disease (bronchial asthma or chronic obstructive pulmonary disease) and for the procedural complications (including conduction abnormalities, bleeding, acute kidney injury, and paravalvular leak). The stroke occurred only in 1 patient; thus, it was omitted from further statistical modeling. The proportional hazards assumption of the Cox models was checked with the Grambsch-Therneau test and diagnostic plots based on Schoenfeld residuals. Interactions between variables in multivariable models were also explored by analyzing multicollinearity for continuous and categorical variables based on the generalized variance inflation factor (GVIF). We used the maximization of log-rank statistics to determine the optimal cut-off values to classify the patients as low or high risk for men and women^15^. The reason for dichotomizing continuous to binary variables was to offer a simple risk classification into “high” versus “low” risk patients for the future use of these parameters in daily medical practice. Kaplan–Meier survival curves were then used to visualize the differences in the overall survival of low- versus high-risk patients and were compared using the log-rank test.

## Results

### Study cohort characteristics and CTL3 parameters

Since the launch of the TAVI Registry in 2010 until the survival censoring on December 31, 2022, 924 patients were enrolled. The availability of preprocedural CT scans at the L3 level enabled us to include 866 patients in our study (429 [49.5%] men and 437 [50.5%] women). There were 778 patients (89.8%, 385 men, 393 women) with hypertension, 375 patients (43.3%, 192 men, 183 women) with type 2 diabetes mellitus, 178 patients (20.6%, 119 men, 59 women) with previously identified coronary heart disease and 267 patients (30.8%, 142 men, 125 women) with history of pulmonary disease. The median follow-up time was 5.89 (3.44–7.89) years with no dropout. We segmented the CT scans at the L3 level to obtain CTL3 parameters for each patient. The baseline characteristics and CTL3 parameters are summarized in Tables 1 and S3.

**Table 1:** Baseline characteristics and CTL3 parameters of the study cohort reported as median (1st, 3rd quartile) or numbers with percentages for the whole cohort and for the patients with and without primary endpoint.

	Total	Alive	Dead
Subject count	866	413 (47.7%)	453 (52.3%)
Age at TAVI [years]	79.72 (74.87, 83.32)	79.33 (74.39, 82.74)	80.36 (75.36, 83.77)
BMI [kg/m^2^]	28.93 (25.92, 32.60)	29.00 (26.04, 32.87)	28.76 (25.76, 32.42)
SMI [cm^2^/m^2^]	44.39 (39.36, 49.84)	44.53 (40.06, 50.10)	44.38 (38.53, 49.24)
IMAT index [cm^2^/m^2^]	8.35 (6.13, 11.45)	7.98 (5.96, 11.32)	8.56 (6.38, 11.51)
VAT index [cm^2^/m^2^]	65.50 (41.26, 87.83)	65.99 (42.26, 85.57)	64.97 (39.00, 88.74)
SAT index [cm^2^/m^2^]	62.90 (44.36, 87.26)	65.27 (47.11, 90.07)	58.84 (42.06, 84.44)
SM density [HU]	30.75 (25.98, 34.91)	30.53 (25.91, 34.57)	30.88 (26.14, 35.13)
IMAT density [HU]	− 67.25 (− 70.97, − 63.94)	− 67.11 (− 71.06, − 63.73)	− 67.40 (− 70.87, − 64.18)
VAT density [HU]	− 95.41 (− 99.82, − 89.89)	− 96.79 (− 100.62, − 90.16)	− 94.31 (− 98.56, − 89.53)
SAT density [HU]	− 99.64 (− 104.99, − 93.40)	− 100.75 (− 106.25, − 95.48)	− 98.93 (− 103.60, − 91.86)
Sex, male	429 (49.5%)	179 (43.3%)	250 (55.2%)
Diabetes mellitus	375 (43.3%)	164 (39.7%)	211 (46.6%)
Hypertension	778 (89.8%)	368 (89.1%)	410 (90.5%)
Coronary heart disease	178 (20.6%)	74 (17.9%)	104 (23.0%)
Respiratory disease	267 (30.8%)	122 (29.5%)	145 (32.0%)
Conduction abnormalities	296 (34.2%)	130 (31.5%)	166 (36.6%)
Bleeding	44 (5.1%)	20 (4.8%)	24 (5.3%)
AKI	6 (0.7%)	2 (0.5%)	4 (0.9%)
PVL	78 (9.0%)	36 (8.7%)	42 (9.3%)

### Selection of informative CTL3 parameters

We assessed the CTL3 parameters for their association with overall survival. First, we calculated Spearman correlation coefficients for the baseline characteristics and the CTL3 parameters to show potential dependencies between the variables. The analysis revealed a strong (0.6 <  ×  <  − 0.6) correlation between VAT density and VAT index (− 0.64) and between VAT density and SAT density (0.79) (Table e S4). We followed with building a univariate Cox proportional hazard model for each of CTL3 parameters; the hazard ratio (HR) with 95% confidence interval (CI) was increased for every 1 HU and 1 cm^2^/m^2^ for density and area index, respectively. The model revealed statistically significant association between the all-cause mortality and the VAT (in HU) (*p* = 0.009; HR = 1.016; 95% CI (1.004–1.029)) and SAT (in HU) (*p* < 0.001; HR = 1.017; 95% CI (1.008–1.026)) densities, and a trend for SMI (in cm^2^/m^2^) (*p* = 0.114; HR = 0.991; 95% CI (0.980–1.002)) (Table S5). We selected the parameters with the strongest association for the multivariable analysis.

We built a Cox proportional hazard model using the informative CTL3 parameters—SMI, VAT density, and SAT density. We adjusted the model for age, sex, and the presence of comorbidities and procedural complications. All parameters were confirmed as significant predictors of survival: SMI (in cm^2^/m^2^) HR 0.986 (95% CI (0.975–0.996); *p* = 0.009), VAT density (in HU) HR 1.015 (95% CI (1.002–1.028); *p* = 0.027) and SAT density (in HU) HR 1.014 (95% CI (1.004–1.023); *p* = 0.005).

### Clinically applicable risk stratification based on SMI, VAT, and SAT densities

We leveraged the maximization of the log-rank technique, a data-driven method for dichotomizing continuous covariates, to find optimal threshold values for stratifying patients into low- and high-risk subgroups based on informative CTL3 predictors. The optimal thresholds of the high-risk subgroups based on SMI, VAT, and SAT densities were < 41.05 cm^2^/m^2^ for men and < 42.44 cm^2^/m^2^ for women, >  − 93.27 HU for men and >  − 95.02 HU for women, and >  − 94.03 HU for men and >  − 96.87 HU for women, respectively. We stratified the patients into subgroups and compared the overall survival using the multivariable Cox proportional hazard model adjusted for age, presence of comorbidities, and procedural complications. The high-risk groups showed an increased mortality risk by 65.1%, 33.6%, and 42.8%, according to SMI, VAT, and SAT densities for males. For female participants, the model showed a trend in increased mortality risk of 30.8% between the groups stratified by VAT density (Table 2).

**Table 2:** Multivariable Cox regression model for overall survival adjusted for age and comorbidities and periprocedural complications determined by the CTL3 predictor thresholds.

CTL3 predictor	Gender	HR (95% CI)	*p*-value
SMI	M	**1.651 (1.258–2.167)**	**< 0.001**
	F	1.125 (0.851–1.488)	0.407
VAT density	M	**1.336 (1.030–1.734)**	**0.029**
	F	1.308 (0.977–1.753)	0.072
SAT density	M	**1.428 (1.092–1.867)**	**0.009**
	F	1.234 (0.913–1.667)	0.171

The overall survival is statistically significantly lower in the high-risk male group and there is a trend in the VAT density group in the female subjects.

Significant values are in bold.

**Figure 2:**
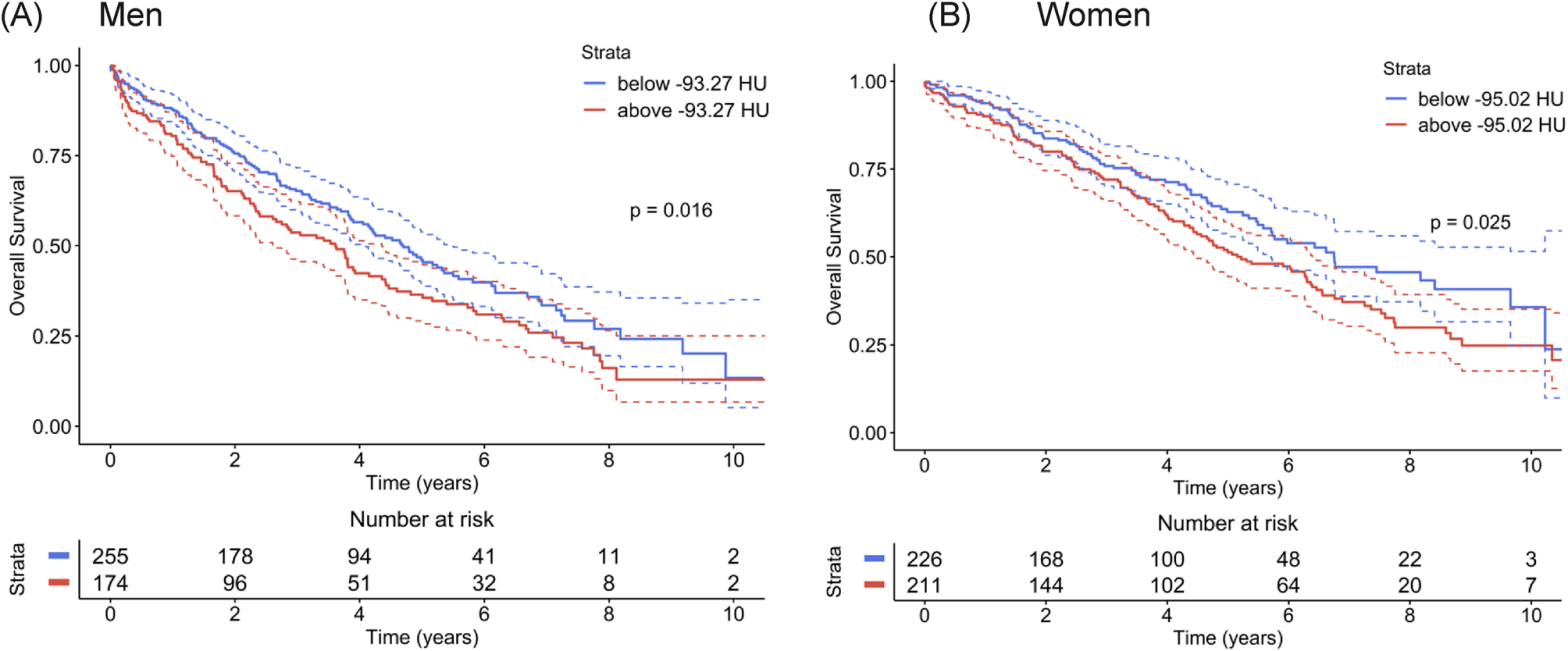
Kaplan–Meier survival curves for categorical VAT density parameter for men (**A**) and women (**B**).

**Figure 3:**
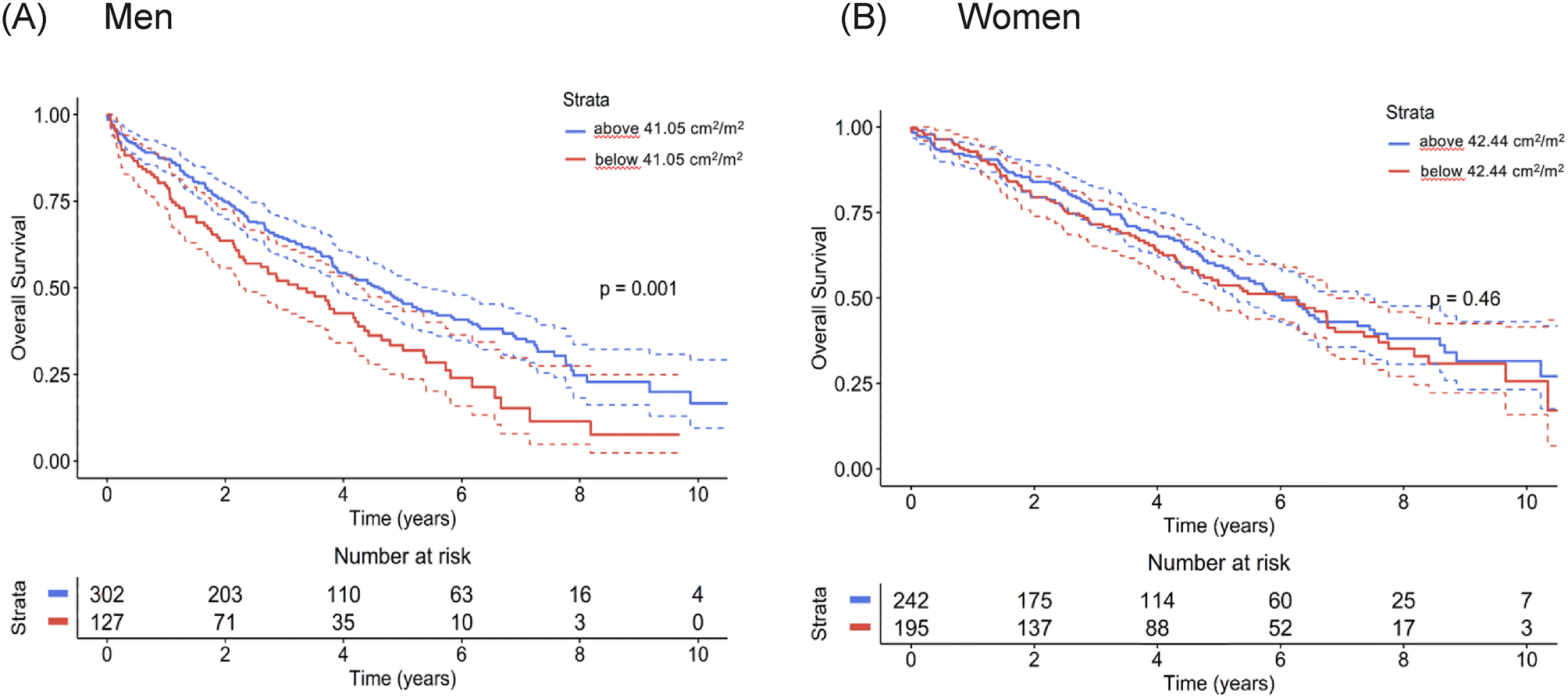
Kaplan–Meier survival curves for categorical SMI parameter for men (**A**) and women (**B**).

**Figure 4:**
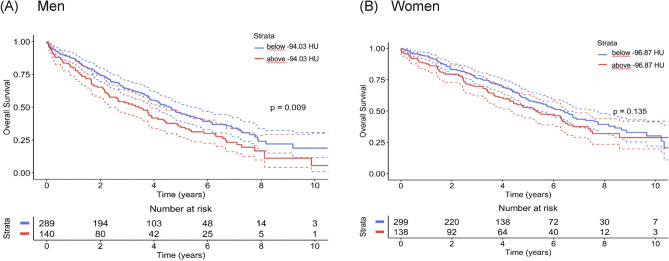
Kaplan–Meier survival curves for categorical SAT density parameter for men (**A**) and women (**B**).

VAT density was identified in the Cox analysis as the possible predictor suitable for estimating overall survival risk in both genders. Thus, besides the Cox models, which reflect the overall hazard ratio during any time point after TAVI, for the final estimation, we performed a Kaplan–Meier survival analysis that demonstrates the mortality risk in individual years after TAVI for high-risk and low-risk groups. The results of this analysis are summarized in Fig. 2, showing standard Kaplan–Meier graphs, and in Table 3. Table 3 can easily be used in clinical reasoning and for discussion with the patients as it offers survival estimates for the individual genders and groups (low- vs. high-risk) in the first five years after the TAVI procedure. Finally, Figs. 3 and 4 depict SMI and SAT density parameters as standard Kaplan–Meier graphs for both men and women.

**Table 3:** Kaplan–Meier survival analysis for categorical VAT density parameter.

Sex	Male		Female	
*p*-value log-rank test	*p* = 0.016		*p* = 0.025	
VAT density cut-off	VAT density below − 93.27 HU for men Survival % (SE)	VAT density above − 93.27 HU for men Survival % (SE)	VAT density below − 95.02 HU for women Survival % (SE)	VAT density above − 95.02 HU for women Survival % (SE)
1-year survival	87.8 (2.1)	80.5 (3.0)	93.8 (1.6)	90.0 (2.1)
2-years survival	75.6 (2.7)	65.1 (3.7)	83.8 (2.5)	80.0 (2.9)
3-years survival	65.2 (3.1)	53.7 (4.0)	75.9 (3.0)	72.0 (3.3)
4-years survival	56.6 (3.4)	42.4 (4.2)	71.3 (3.3)	62.0 (3.6)
5-years survival	46.1 (3.6)	36.4 (4.1)	62.8 (3.8)	51.3 (3.8)

## Discussion

TAVI represents a minimally invasive procedure that enables the replacement of the dysfunctional aortic valve using a trans-catheter approach without the need for open-chest operation^1^. The alternative is the surgical aortic valve replacement (SAVI), a highly invasive procedure that is rarely suitable for elderly patients, in whose aortic valve dysfunction is most commonly diagnosed^16^. Patients indicated for TAVI are thus high risk per se (compared to SAVI patients), and identifying novel tools to stratify individual risks further is of utmost importance.

Analyzing the body composition parameters using cutting-edge computer vision AI methods opens a promising venue for the stratification of individual risks^4–6^. The body composition is a combination of the lifestyle and the presence of various diseases, and, at the same time, the body composition itself may predispose individuals to developing other conditions, such as hypertension or diabetes mellitus^17^. The commonly used anthropometric parameters, such as height, weight, BMI, or waist circumference, are easily accessible but provide only limited informative value. The CT scans are available for all patients indicated for TAVI; the scans can be rapidly analyzed using AI-driven methods to derive the body composition parameters to better inform the clinicians about the individual’s complications or mortality risks, enabling personalized patient care.

Despite the availability of the complete spiral CT of the thorax, we limited our analysis to the L3 cross-section because the L3 scans were previously shown to correlate with the overall muscle condition^8,18–23^. We analyzed the surrounding adipose tissue areas and densities in a similar framework by applying automatic tissue segmentation in a large cohort from the TAVI registry from Hospital AGEL Třinec-Podlesí. We show that the CT-derived parameters are associated with the overall survival of the patients in a sex-dependent manner—SMI, VAT, and SAT densities were associated with the overall survival in men, while in women, there was an observed trend for VAT density.

### Skeletal muscle index (SMI)

From selected CTL3 predictors, SMI is used to evaluate sarcopenia. Sarcopenia represents a common comorbidity in elderly individuals that further affects their quality of life, morbidity, and mortality^24^. The prevalence of sarcopenia is very high and increases with age^25^; despite this fact, it is still broadly underdiagnosed, and its significance is commonly underestimated. Remarkably, patients indicated for TAVI procedure represent a high-risk elderly population with severe aortic valve stenosis that is not eligible for SAVI—this patients’ group is thus more prone to present with sarcopenia than SAVI patients. Determining the extent of sarcopenia in this patient group is therefore reasonable and may prove beneficial in terms of further preoperative management and estimation of peri- and post-operative complications.

SMI has already been used in several previous studies that focused on the mortality of patients after TAVI^8,11,23,26–28^. We have shown the association of SMI with all-cause mortality in the subgroup of men indicated for TAVI. Most of the similar studies showed associations of SMI with all-cause mortality^8,11,23,26^, prolonged hospital stay ^27,28^, or the occurrence of adverse events^11^ in sarcopenic patients with low SMI by using sex-specific cut-off values for men and women. Nevertheless, some studies showed associations with mortality only when SMI combined with skeletal muscle density^21^ or did not prove an association with mortality at all^27,28^.

These variable results of individual studies are mainly based on the methodological issues arising from the absence of an internationally recognized threshold for SMI. Most of the studies^8,21,26^ used the sex-specific cut-off values of 55 and 39 cm^2^/m^2^ for men and women, respectively, that are derived from the study by Baumgartner et al. from 1998^29^, while some were comparing highest and lowest SMI quartiles^11^, sex-specific tertiles^23^ or two times standard deviation from the gender-specific references^8^.

For our study, we used cut-off values for SMI (and for all other parameters) by using the maximization of the log-rank score method^15^; as the study by Baumgartner et al. included general Hispanic and non-Hispanic population from New Mexico, its transferability to the Central-European mainly non-Hispanic population of the Czech Republic is not validated. We have shown higher HR (1.651 (1.258–2.167)) in sarcopenic males for overall mortality, an effect similar to those observed in other studies^6^. Our study contrasts the study by Mourik et al., who showed an association of low SMI with mortality in a subgroup of women^23^; nevertheless, the authors used different cut-off values (tertiles).

### Visceral and subcutaneous adipose tissue (VAT and SAT) density

The next studied CTL3 predictors evaluated the visceral and subcutaneous adipose tissue quality according to their attenuation (so-called density).

Besides sarcopenia related to skeletal muscles, accumulation of adipose tissue leading to obesity is known to alter the patient’s prognosis in various settings significantly—the presence of obesity is a known risk factor for increased CV mortality^17^, nevertheless, data are showing the “obesity paradox,” i.e., the fact that in some situation presence of obesity may be beneficial, as also shown in obese TAVI patients using BMI^7^. Adipose tissue can be accumulated in the visceral compartment (more common in men) or subcutaneous compartment (more common in women). Similarly to the previous study^8^, men presented with a higher VAT index than women, and women presented with a higher SAT index than men also in our study. Nevertheless, the amount of fat indexed for body height did not affect all-cause mortality in our study, similar to the study by Shibata et al.^10^. On the other hand, lower VAT and SAT area (not indexed to height) in the study by Foldyna predicted worse outcomes after TAVI^30^.

Interestingly, we have observed that lower VAT and SAT density values predict better survival in men. At the same time, there was only a trend for better survival in women with lower VAT density values. CT density expressed in HU reflects the composition of the tissue—for the adipose tissue, studies suggest that its attenuation is affected by the size of the adipocytes (lower attenuation was associated with larger adipocyte size^31^) and results from the Framingham study shows that lower values of VAT density are associated with more cardiometabolic risk factors^32^ and with altered adipokines production that is more favorable to increase cardiometabolic risk^33^. Thus, differently-sized adipocytes seem to be differently hormonally active, and while lower VAT densities predict increased cardiovascular mortality in the general population, in our study we have observed the opposite trend, thus partially confirming the presence of “obesity paradox” in the elderly patients indicated for TAVI. Similar results for higher SAT density predicting higher overall mortality were observed also in the study by Foldyna^30^.

### Study strengths and limitations

Within the study, we used semi-automated evaluation of the CT scans by artificial intelligence software based on the neuronal nets function, which provides standardized results not affected by individual manual measurements of the muscle and fat areas and densities. Another strength of the study is the large sample size and long follow-up period, which also enabled the subanalysis to include gender (gender plays a substantial role in cardiovascular studies, and our study confirms that there are significant differences between both genders). Study results can also be easily implemented into clinical practice. Rigorous statistical processing also ensures good reproducibility.

Study limitations include the mono-centricity and retrospective evaluation of the CT scans, which led to the non-involving of some patients from the Registry due to missing or low-quality CT scans.

## Conclusion and future direction

Within our study, we have confirmed and further expanded the results from previous studies showing that the quality of the visceral adipose tissue (expressed as VAT density), the quality of subcutaneous adipose tissue (expressed as SAT density), and the presence of sarcopenia (expressed as SMI) predicts higher all-cause mortality in men undergoing TAVI procedure in the Central-European population. In women, only the quality of the visceral adipose tissue tends to predict all-cause mortality. These results can be easily implemented into clinical practice as automatic artificial intelligence-based software was used, enabling the reproducibility of the results and fast analysis of preoperative CT scans routinely performed before the TAVI procedure. Our results shall also stimulate interventional studies focusing on exercise or nutritional interventions in the TAVI population to diminish the effect of sarcopenia, malnutrition, or obesity.

### Supplementary Information

Supplementary Information 1.

Supplementary Information 2.

Supplementary Information 3.

Supplementary Information 4.

Supplementary Information 5.

## Data Availability

The datasets used and/or analysed during the current study are available from the corresponding author on reasonable request.
