# Publisher Correction: Sarcopenia and adipose tissue evaluation by artificial intelligence predicts the overall survival after TAVI

**DOI:** 10.1038/s41598-024-61601-6

**Published:** 2024-05-10

**Authors:** Matej Pekař, Otakar Jiravský, Jan Novák, Piotr Branny, Jakub Balušík, Daniel Daniš, Jan Hečko, Marek Kantor, Robert Prosecky, Lubomir Blaha, Radek Neuwirth

**Affiliations:** 1 Hospital AGEL Třinec-Podlesí, Konská 453, 739 61 Třinec, Czech Republic; 2 Department of Physiology, Faculty of Medicine, Masaryk University, Kamenice 5, 625 00 Brno, Czech Republic; 3 2nd Department of Internal Medicine, St. Anne’s University Hospital in Brno, Pekařská 53, 656 91 Brno, Czech Republic; 4 Faculty of Medicine, Masaryk University, Kamenice 5, 625 00 Brno, Czech Republic; 5 Faculty of Medicine, Palacky University, Krizkovskeho 511/8, 779 00 Olomouc, Czech Republic; 6 The Jackson Laboratory for Genomic Medicine, 10 Discovery Drive, Farmington, CT 06032 USA; 7 Faculty of Electrical Engineering and Computer Science, VSB - Technical University of Ostrava, 17. Listopadu Street 2172/15, 708 00 Ostrava, Czech Republic

Correction to: *Scientific Reports* 10.1038/s41598-024-59134-z, published online 17 April 2024

In the original version of this Article Matej Pekař was incorrectly affiliated with ‘Faculty of Medicine, Masaryk University, Kamenice 5, 625 00 Brno, Czech Republic’. The correct affiliations for Matej Pekař are listed below.

Hospital AGEL Třinec-Podlesí, Konská 453, 739 61 Třinec, Czech Republic.

Department of Physiology, Faculty of Medicine, Masaryk University, Kamenice 5, 625 00 Brno, Czech Republic.

The original Article has been corrected.

