# Supplementary material for: Sarcopenia and adipose tissue evaluation by artificial intelligence predicts the overall survival after TAVI
Source: Sci Rep. 2024 Apr 17;14:8842. doi: 10.1038/s41598-024-59134-z (PMC11024085; doi:10.1038/s41598-024-59134-z)
Supplement: Supplementary file 1 — Supplementary Information 1. [file 41598_2024_59134_MOESM1_ESM.docx]

**Sarcopenia and adipose tissue evaluation by artificial intelligence predicts the overall survival after TAVI**

**Table S1:** TAVI procedure basic characteristics

| **Characteristic** | **Values** |
| --- | --- |
| procedural time  (minutes, median, 1 st , 3 rd quartile) | 60 (45, 85) |
| contrast used volume  (ml, median, 1 st , 3 rd quartile) | 180 (150, 220) |
| sheath (french, median, 1 st , 3 rd quartile) | 18 (14,18) |
| balloon predilatation valvuloplasty (n) | 714 |
| balloon predilatation diameter (mm, n) | 16 2  18 63  20 175  22 206  23 125  24 42  25 96  26 4  28 1 |
| balloon postdilatation valvuloplasty (n) | 120 |
| balloon postdilatation diameter (mm, n) | 18 1  20 18  22 22  23 19  24 19  25 32  26 3  28 5  30 1 |
| anesthesia (type, n) | General 435  Local 431 |
| access (type, n) | Femoral percutaneous 687  Femoral surgical 2  Subclavian 72  Direct aortic. 57  Teansapical 48 |
