# Supplementary material for: Sarcopenia and adipose tissue evaluation by artificial intelligence predicts the overall survival after TAVI
Source: Sci Rep. 2024 Apr 17;14:8842. doi: 10.1038/s41598-024-59134-z (PMC11024085; doi:10.1038/s41598-024-59134-z)
Supplement: Supplementary file 2 — Supplementary Information 2. [file 41598_2024_59134_MOESM2_ESM.docx]

**Sarcopenia and adipose tissue evaluation by artificial intelligence predicts the overall survival after TAVI**

**Table S2:** TAVI peri-procedural complications

| **Complication** | Stroke | Conduction abnormalities | Bleeding | Acute kidney injury | Paravalvular leak |
| --- | --- | --- | --- | --- | --- |
| **n** | 1 | 296 | 44 | 6 | 78 |
