# Supplementary material for: Sarcopenia and adipose tissue evaluation by artificial intelligence predicts the overall survival after TAVI
Source: Sci Rep. 2024 Apr 17;14:8842. doi: 10.1038/s41598-024-59134-z (PMC11024085; doi:10.1038/s41598-024-59134-z)
Supplement: Supplementary file 3 — Supplementary Information 3. [file 41598_2024_59134_MOESM3_ESM.docx]

**Sarcopenia and adipose tissue evaluation by artificial intelligence predicts the overall survival after TAVI**

**Table S3:** Baseline characteristics and CTL3 parameters of the study cohort reported as median (1 st , 3 rd quartile) or numbers with percentages for the whole cohort and for the patients with and without primary endpoint divided into male versus female subgroups.

|  | **whole cohort** |  |
| --- | --- | --- |
|  | **Male** | **Female** |
| **Subject count** | 429 (49.5%) | 437 (50.5%) |
| **Age at TAVI [years]** | 78.36 (73.37, 82.98) | 80.5 (76.58, 83.58) |
| **BMI [kg/m^2^]** | 27.89 (25.56, 31.18) | 29.94 (26.30, 34.17) |
| **SMI [cm^2^ /m^2^]** | 45.96 (40.13, 51.66) | 43.32 (38.94, 47.71) |
| **IMAT index [cm^2^ /m^2^]** | 7.65 (5.72, 10.27) | 9.11 (6.58, 12.86) |
| **VAT index [cm^2^ /m^2^]** | 70.48 (43.18, 93.89) | 60.3 (39.27, 81.17) |
| **SAT index [cm^2^ /m^2^]** | 46.87 (35.75, 59.45) | 83.73 (65.27, 106.87) |
| **SM density [HU]** | 32.13 (28.00, 36.25) | 29.07 (24.38, 33.61) |
| **IMAT density [HU]** | -67.43 (-71.28, -63.98) | -67.09 (-70.68, -63.79) |
| **VAT density [HU]** | -95.36 (-100.03, -89.53) | -95.42 (-99.56, -90.20) |
| **SAT density [HU]** | -98.31 (-103.52, -91.86) | -100.91 (-106.25, -95.32) |
| **Diabetes mellitus** | 192 (44.8%) | 183 (41.9%) |
| **Hypertension** | 385 (89.7%) | 393 (89.9%) |
| **Coronary heart disease** | 119 (27.7%) | 59 (13.5%) |
| **Respiratory disease** | 142 (33.1%) | 125 (28.6%) |
| **Conduction abnormalities** | 174 (40.6%) | 122 (27.9%) |
| **Bleeding** | 20 (4.7%) | 24 (5.5%) |
| **AKI** | 6 (1.4%) | 0 (0%) |
| **PVL** | 46 (10.7%) | 32 (7.3%) |

|  | **Male - alive** | **Male - dead** |
| --- | --- | --- |
| **Subject count** | 179 (20.67 %) | 250 (28.87 %) |
| **Age at TAVI [years]** | 77.01 (73.30, 82.61) | 78.84 (74.01, 83.43) |
| **BMI [kg/m^2^]** | 28.23 (25.56, 31.40) | 27.73 (25.51, 30.96) |
| **SMI [cm^2^ /m^2^]** | 46.68 (41.02, 52.07) | 45.33 (39.02, 51.63) |
| **IMAT index [cm^2^ /m^2^]** | 7.16 (5.44, 9.87) | 7.91 (6.00, 10.45) |
| **VAT index [cm^2^ /m^2^]** | 71.85 (44.83, 93.62) | 68.94 (38.44, 94.19) |
| **SAT index [cm^2^ /m^2^]** | 48.22 (38.36, 60.53) | 46.15 (34.76, 58.77) |
| **SM density [HU]** | 32.11 (27.71, 36.43) | 32.13 (28.00, 36.25) |
| **IMAT density [HU]** | -67.09 (-70.62, -63.98) | -68.01 (-71.53, -63.97) |
| **VAT density [HU]** | -96.64 (-100.74, -90.05) | -94.36 (-98.95, -88.70) |
| **SAT density [HU]** | -99.04 (-105.07, -93.86) | -97.09 (-102.79, -90.04) |
| **Diabetes mellitus** | 68 (38.0%) | 124 (49.6%) |
| **Hypertension** | 159 (88.8%) | 226 (90.4%) |
| **Coronary heart disease** | 48 (26.8%) | 71 (28.4%) |
| **Respiratory disease** | 58 (32.4%) | 84 (33.6%) |
| **Conduction abnormalities** | 74 (41.3%) | 100 (40.0%) |
| **Bleeding** | 7 (3. 9%) | 13 (5.2%) |
| **AKI** | 2 (1.1%) | 4 (1.6%) |
| **PVL** | 19 (10.6%) | 27 (10.8%) |

|  | **Female - alive** | **Female - dead** |
| --- | --- | --- |
| **Subject count** | 234 (27.02 %) | 203 (23.44 %) |
| **Age at TAVI [years]** | 79.92 (75.28, 82.83) | 81.52 (77.43, 84.21) |
| **BMI [kg/m^2^]** | 29.54 (26.56, 34.55) | 30.12 (26.17, 33.83) |
| **SMI [cm^2^ /m^2^]** | 43.45 (39.25, 48.10) | 43.12 (38.43, 47.13) |
| **IMAT index [cm^2^ /m^2^]** | 8.90 (6.44, 12.32) | 9.27 (7.06, 13.26) |
| **VAT index [cm^2^ /m^2^]** | 61.44 (39.56, 79.44) | 59.37 (39.00, 83.44) |
| **SAT index [cm^2^ /m^2^]** | 83.53 (65.75, 106.53) | 83.73 (63.83, 108.16) |
| **SM density [HU]** | 28.99 (23.92, 33.71) | 29.32 (24.60, 33.51) |
| **IMAT density [HU]** | -67.16 (-71.08, -63.58) | -67.06 (-69.91, -64.20) |
| **VAT density [HU]** | -98.87 (-100.61, -90.31) | -94.31 (-98.00, -89.89) |
| **SAT density [HU]** | -101.81 (-107.07, -95.90) | -99.99 (-104.87, -93.90) |
| **Diabetes mellitus** | 96 (41.0%) | 87 (42.9%) |
| **Hypertension** | 209 (89.3%) | 184 (90.6%) |
| **Coronary heart disease** | 26 (11.1%) | 33 (16.3%) |
| **Respiratory disease** | 64 (27.4%) | 61 (30.0%) |
| **Conduction abnormalities** | 56 (23.9%) | 66 (32.5%) |
| **Bleeding** | 13 (5.6%) | 11 (5.4%) |
| **AKI** | 0 (0%) | 0 (0%) |
| **PVL** | 17 (7.3%) | 15 (7.4%) |
