# Supplementary material for: Sarcopenia and adipose tissue evaluation by artificial intelligence predicts the overall survival after TAVI
Source: Sci Rep. 2024 Apr 17;14:8842. doi: 10.1038/s41598-024-59134-z (PMC11024085; doi:10.1038/s41598-024-59134-z)
Supplement: Supplementary file 4 — Supplementary Information 4. [file 41598_2024_59134_MOESM4_ESM.docx]

**Sarcopenia and adipose tissue evaluation by artificial intelligence predicts the overall survival after TAVI**

**Table S4:** Spearman correlation coefficients for the baseline characteristics and CTL3 parameters.

|  | **Age** | **Height** | **BMI** | **BSA** | **SMI** | **IMAT index** | **VAT index** | **SAT index** | **SM density** | **IMAT density** | **VAT density** | **SAT density** |
| --- | --- | --- | --- | --- | --- | --- | --- | --- | --- | --- | --- | --- |
| **Age** | 1 |  |  |  |  |  |  |  |  |  |  |  |
| **Height** |  | 1 |  |  |  |  |  |  |  |  |  |  |
| **BMI** |  |  | 1 |  |  |  |  |  |  |  |  |  |
| **BSA** | -0.37 | **0.68** | **0.6** | 1 |  |  |  |  |  |  |  |  |
| **SMI** |  |  |  |  | 1 |  |  |  |  |  |  |  |
| **IMAT index** |  |  | 0.48 |  |  | 1 |  |  |  |  |  |  |
| **VAT index** |  |  | 0.59 | 0.5 |  | 0.31 | 1 |  |  |  |  |  |
| **SAT index** |  | -0.47 | **0.64** |  |  | 0.53 | 0.28 | 1 |  |  |  |  |
| **SM density** |  |  |  |  |  | -0.59 | -0.36 | -0.33 | 1 |  |  |  |
| **IMAT density** |  |  |  |  | 0.35 | -0.44 |  |  |  | 1 |  |  |
| **VAT density** |  |  | -0.42 | -0.33 |  | -0.33 | **-0.64** | -0.33 |  | 0.55 | 1 |  |
| **SAT density** |  |  | -0.48 | -0.30 |  | -0.4 | -0.42 | -0.55 |  | 0.59 | **0.79** | 1 |

The values with strong correlation (0.6<x<-0.6) use boldface and we do not present values in range (-0.3, 0.3). The strongest correlation of 0.79 was observed between SAT and VAT densities.

Abbreviations: BMI - body mass index, BSA - body surface area (DuBois formula), SMI - skeletal muscle index, IMAT - intramuscular adipose tissue, VAT - visceral adipose tissue, SAT - subcutaneous adipose tissue, SM - skeletal muscle
