# Supplementary material for: Sarcopenia and adipose tissue evaluation by artificial intelligence predicts the overall survival after TAVI
Source: Sci Rep. 2024 Apr 17;14:8842. doi: 10.1038/s41598-024-59134-z (PMC11024085; doi:10.1038/s41598-024-59134-z)
Supplement: Supplementary file 5 — Supplementary Information 5. [file 41598_2024_59134_MOESM5_ESM.docx]

**Sarcopenia and adipose tissue evaluation by artificial intelligence predicts the overall survival after TAVI**

**Table S5:** Univariate Cox analysis

|  | **HR (95% CI)** | **P value** |
| --- | --- | --- |
| **SMI** | 0.991 (0.980 - 1.002) | 0.114 |
| **VAT density** | 1.016 (1.004 - 1.029) | 0.009 |
| **SAT density** | 1.017 (1.008 - 1.026) | <0.001 |

Univariate Cox analysis was performed for individual CTL3 parameters, revealing statistically significant results for VAT and SAT density and trend for SMI.

Abbreviations: SMI - skeletal muscle index, VAT - visceral adipose tissue, SAT - subcutaneous adipose tissue, , HR - hazard ratio, CI - confidence interval
